# Does childhood maltreatment influence Chinese preschool education college students’ depression and anxiety? Evidence from a latent class analysis

**DOI:** 10.3389/fpsyg.2024.1341344

**Published:** 2024-03-28

**Authors:** Shengkai Ji, Chen Chen

**Affiliations:** ^1^College of Child Development and Education, Zhejiang Normal University, Hangzhou, China; ^2^Center for Educational Science and Technology, Beijing Normal University, Zhuhai, China

**Keywords:** childhood maltreatment, depression, preschool education college students, anxiety, latent class analysis

## Abstract

**Introduction:**

Preschool teachers’ mental health may be a critical factor in their job performance, which in turn can influence the quality of early childhood education. However, little is known about its development. Childhood maltreatment, as a chronic form of childhood stress, may influence later mental health development. Although large, comprehensive research has been undertaken on childhood maltreatment and mental health, the associations between these two variables need further exploration. This study aims to describe childhood maltreatment based on person-centered approaches in Chinese preschool education college students, and to examine the differences in depression and anxiety within subgroups of childhood maltreatment.

**Methods:**

A total of 1,218 Chinese preschool education college students participated in this study, and the data analysis was based on unconditional and conditional latent class analysis.

**Results:**

The results showed that the participants were divided into 5 profiles: a non-maltreatment profile; a profile of emotional abuse and emotional and physical neglect; a profile of physical neglect; a profile of emotional neglect; and a profile of physical and emotional neglect. Students with multiple subtypes of maltreatment had the highest levels of depression and anxiety.

**Discussion:**

Universities should consider both childhood maltreatment and profiles of childhood maltreatment when training preschool education college students.

## Introduction

Mental health is a critical global public health issue that affects individuals’ work performance and well-being (Merlin et al., 2022; Vesely et al., 2024), and depression and anxiety are the two most common mental health problems (World Health Organization [WHO], 2020), which may be contributed to by past experiences. Childhood maltreatment as an adverse childhood experience may affect individuals’ perceptions of themselves and others (e.g., biased internal working models; Gong and Chan, 2018), which may contribute to the negative impact on later mental health status (Chen and Qin, 2020a; Humphreys et al., 2020; Chauhan et al., 2023) and suicidal behavior (e.g., Baldini et al., 2023). Although the associations between childhood maltreatment and depression and anxiety have been examined in a growing number of studies (Javakhishvili and Widom, 2021), these relationships still need further exploration, particularly the role of childhood maltreatment profiles.

Furthermore, the college period is the beginning of adulthood and it may be challenging for individuals due to psychological and environmental changes that may contribute to maladaptive problems, such as depression and anxiety (Çalışkan et al., 2023; Chen et al., 2024). Moreover, college students majoring in early childhood education are different from other types of college students due to their future work with young children, and depression and anxiety in preschool teachers reduce the quality of early childhood education (e.g., teacher-child interactions; Soininen et al., 2023). Therefore, the current study aims to explore the relationship between childhood maltreatment and depression and anxiety, taking into account the profiles of childhood maltreatment in Chinese preschool education college students, which may provide suggestions for their specific training.

### Childhood maltreatment profiles

Childhood maltreatment, as an adverse childhood experience, includes acts of commission and omission against a child. Physical abuse, emotional abuse, and sexual abuse are three major subtypes of childhood maltreatment that represent acts of commission; physical neglect, emotional neglect, and educational neglect are three major subtypes of acts of omission (U.S. Department of Health and Human Services, 2017). Childhood maltreatment can affect an individual’s mental health. For example, Chen and Qin (2020a) reported that emotional abuse was positively associated with social anxiety in adolescents. Zheng et al. (2020) found that childhood maltreatment was positively associated with depression in college students. Additionally, Nelson et al. (2017) explored the relationship between childhood maltreatment and adult depression based on a meta-analysis and found that nearly half of patients with depression reported a history of childhood maltreatment.

Previous studies have explored the relationship between childhood maltreatment and mental health (e.g., depression and anxiety) under the assumption of homogeneity among subjects, whereas not all individuals have the same characteristics of childhood maltreatment. These gaps in previous studies may create an urgency to consider profiles of childhood maltreatment. Person-centered approaches, characterized by capturing heterogeneity within a sample, provide a new perspective for examining variables that may provide a better understanding of individual differences. Latent class analysis (LCA), as a person-centered approach, is used to classify individuals into subgroups based on individual characteristics (Collins and Lanza, 2009), where individuals within a subgroup are similar to but different from those in the other groups (Muthén and Muthén, 2017).

Some studies have explored the profiles of childhood maltreatment based on person-centered approaches. For instance, Nooner et al. (2010) found that there were four classes or profiles of physical and sexual abuse among American preadolescents, including a no abuse subgroup, a high physical abuse and low sexual abuse subgroup, a no physical abuse and moderate sexual abuse subgroup, and a high physical and sexual abuse subgroup. Similarly, Armour et al. (2013) reported that 2,980 young adults (age 24) in Denmark were divided into four distinct abuse profiles, including a non-abused group, an emotionally abused group, a sexually abused group, and a group with multiple types of maltreatment. Meanwhile, Katz et al. (2021) found three subgroups of childhood maltreatment among 5,872 youths, including a subgroup of neglect and adverse parental behaviors, a subgroup of physical abuse, and a subgroup of sexual abuse. Additionally, Zhang et al. (2022) reported that 10,515 adolescents were divided into two subgroups based on childhood maltreatment, including no maltreatment and multiple maltreatment.

These findings suggest that individuals may have experienced different types, severity, or timing of childhood maltreatment and that individual characteristics may influence the abuse experienced. Although several studies have examined profiles of childhood maltreatment in Western samples, this research topic needs further exploration in the Chinese cultural context. Specifically, traditional Chinese culture emphasizes hierarchy within the family, which may influence modern Chinese parenting beliefs, and in turn, contribute to different characteristics of childhood maltreatment (Chen and Qin, 2020b). Moreover, previous studies have not considered the severity of childhood maltreatment when identifying its profiles. Therefore, this study aims to identify the profiles of childhood maltreatment in Chinese preschool education college students based on both the subtype and severity of it, and it hypothesizes that childhood maltreatment is heterogeneous, and has multiple profiles.

### Childhood maltreatment, depression and anxiety

Childhood maltreatment may have an impact on depression and anxiety in individuals, based on a growing body of research. For instance, Javakhishvili and Widom (2021) showed that childhood maltreatment positively predicted anxiety and depression in young Americans. Sun et al. (2022) reported that childhood maltreatment had indirect effects on depression in 2310 Chinese adolescents. These associations were also confirmed in a meta-analysis (Humphreys et al., 2020). These previous studies assumed that the entire sample of participants had experienced the same childhood maltreatment, which may ignore the effects of individual differences (Collins and Lanza, 2009). Meanwhile, these previous studies may attribute the effects of childhood maltreatment on individual development to a single subtype rather than the cumulative experience of maltreatment, which may not clearly delineate the complexity of childhood maltreatment with high rates of overlap among subtypes.

Moreover, the role of profiles of childhood maltreatment in the associations between childhood maltreatment and depression and anxiety has been explored in previous studies. For instance, Katz et al. (2021) reported that depression differed based on three classes of maltreatment, specifically, individuals belonging to the sexual maltreatment profile reported the highest levels of depression at Waves 1 and 3, and individuals belonging to the physical maltreatment profile reported the highest levels of depression at Wave 2. Zhang et al. (2022) also confirmed these relationships among Chinese adolescents. These findings suggest that profiles of childhood maltreatment may be an important factor in the associations between childhood maltreatment and later mental health status.

Although the role of profiles of childhood maltreatment has been studied, this issue is still unclear in the Chinese cultural context. Moreover, based on the theory of developmental psychopathology, individuals who have suffered accumulated trauma may have the most severe impairment in later development, while the relationships between the accumulation of childhood maltreatment and mental health status need further exploration. Therefore, this study attempts to examine the role of profiles of childhood maltreatment in the associations between childhood maltreatment and depression and anxiety in Chinese college students of preschool education. This study hypothesizes that depression and anxiety are different based on profiles of childhood maltreatment, and individuals who belong to multiple subtypes of childhood maltreatment have the highest level of depression and anxiety.

### About the current study

Early childhood education (ECE), an important early childhood experience, is a worldwide factor for the later development of individuals (Baker-Henningham et al., 2009; Burchinal, 2018), and how to promote the quality of ECE has been an important issue for nations. Preschool teachers are an important component of the quality of ECE (Peele and Wolf, 2021) and while large and comprehensive studies have focused on preschool teachers, such as work stress, few studies have explored the role of negative childhood experiences in the training of preschool teachers.

Moreover, China has focused on promoting the quality of ECE in recent decades by improving the skills of preschool education college students, and childhood experiences may have an impact in this regard. Meanwhile, China has been influenced by Confucian culture for a long time, which may contribute to different social and cultural characteristics from Western countries, and in turn, lead to different characteristics of child discipline. Additionally, the role of childhood maltreatment profiles in the relationship between childhood maltreatment and depression and anxiety is not clear. Therefore, we are guided by developmental psychopathology theory which posits that adverse experiences may influence later development (Toth and Cicchetti, 2013), and nonlinear dynamic systems theory, which posits that individual differences should be taken into consideration when delineating individual development (Thelen and Smith, 1994). The current study aims to (1) explore profiles of childhood maltreatment, and (2) describe the role of profiles of childhood maltreatment in the relationship between childhood maltreatment and depression and anxiety among Chinese college students in preschool education. We hypothesize that (1) childhood maltreatment in Chinese preschool education college students is heterogeneous, and has multiple profiles, (2) depression and anxiety are different based on profiles of childhood maltreatment, and individuals who belong to multiple subtypes of childhood maltreatment have the highest level of depression and anxiety.

## Methodology

### Participants

Participants were recruited from the Chinese Longitudinal Study of Preschool Teacher Students Development (CLSPTSD), which aimed to explore the development trajectories and development factors of pre-service preschool teachers in the Chinese cultural context. Three provinces were recruited in this study, including Zhejiang Province, Jiangsu Province, and Liaoning Province, and 1,218 preschool education college students were randomly selected to participate in this study. Among the participants, 95.4% were women, and the mean age was 19.19 years old (SD =0.94). The demographic information is presented in Table 1.

**Table 1:** Demographic information on the participants (*n* = 1,218).

		*N* (%)
Age		19.19 ± 0.94
Gender	Girl	1,162 (95.4)
	Boy	56 (4.6)
Only child	Yes	605 (49.7)
	No	613 (50.3)
Left-behind children	Yes	99 (8.1)
	No	1,119 (91.9)
Father’s education	Primary and below	146 (12.0)
	Secondary school	541 (44.4)
	High school	302 (24.8)
	College school	122 (10.0)
	Bachelor’s degree	98 (8.0)
	Master’s degree and above	9 (0.7)
Mother’s education	Primary and below	200 (16.4)
	Secondary school	537 (44.1)
	High school	281 (23.0)
	College school	121 (9.9)
	Bachelor’s degree	72 (5.9)
	Master’s degree and above	7 (0.6)
Family month-income	$145 and below	39 (3.2)
	$145 ~ $435	182 (14.9)
	$435 ~ $725	289 (23.7)
	$725 RMB ~ $1,450	445 (36.5)
	$1,450 ~ $4,350	232 (19.0)
	$4,350 and above	31 (2.5)

### Measures

#### Childhood trauma questionnaire-short form (CTQ-SF)

The CTQ-SF is a 28-item self-report instrument used to assess childhood maltreatment before the age of 18 years. It was compiled by Bernstein et al. (2003) and the Chinese version of it was developed by Zhao et al. (2005). The questionnaire has five subscales: physical neglect (e.g., *no food*), emotional neglect (e.g., *feeling loved*), physical abuse (e.g., *being hit hard*), emotional abuse (e.g., *being yelled at*), and sexual abuse (e.g., *watching sexually explicit videos*). Participants respond to each item on a five-point scale (1 = *never* to 5 = *almost always*), with higher scores indicating more childhood maltreatment. Due to cultural differences and the uniqueness of the participants’ ages, this study selected four subscales of the CTQ-SF to collect data such as physical and emotional neglect, and physical and emotional abuse. The sexual abuse subscale was not administered in this study due to its characteristics and the aforementioned cultural differences. Specifically, sexual abuse may represent the metaphrenia in sex and it is different from other subtypes of childhood maltreatment. Talking about sex may be taboo in traditional Chinese culture, which may influence modern Chinese beliefs, and in turn, contribute to inaccurate results of sexual abuse. The mean scores of these four subscales were used for data analysis. The Chinese version of the CTQ-SF has been used among college students (Liu et al., 2021), and the Cronbach’s alpha of these four subscales and the total scale in this study were 0.89, 0.70, 0.78, 0.71, and 0.89, respectively.

#### The center for epidemiologic studies-depression scale (CES-D)

The CES-D, a 20-item self-report rating scale, was developed by Radloff (1977), aiming to measure depression in college students in preschool education. Each item is rated on a four-point Likert scale (1 = *rarely or less than a day* to 4 = *most of the time*), with higher scores indicating more depression. The Chinese version of the CES-D has good reliability and validity (Ye et al., 2018). The Cronbach’s alpha for the CES-D in this study was 0.84.

#### Self-rating anxiety scale (SAS)

The SAS was developed by Zung (1971), aiming to measure anxiety, and the Chinese version was developed by Liu et al. (1997). This instrument consists of 20 items (e.g., *Felt fear without reasons*) and uses a four-point Likert scale (1 = *never or very rarely* to 4 = *very often or always*), with higher scores indicating greater anxiety. The Chinese version of the SAS had already been used with Chinese college students (Zhao et al., 2021), and the Cronbach’s alpha in this study was 0.91.

### Procedure

The data collection procedure for this study was carried out in several steps. First, four normal universities from different areas of China were selected by convenience sampling according to their different development of early childhood education, and three of them agreed to participate in this study. Next, classes within the universities were selected by random sampling, and then questionnaires were sent to students in preschool education. Specifically, the authors sent online questionnaires to all college students in preschool education at these three universities. Finally, 1,218 students participated in this study, and they signed an online informed consent form before answering the questionnaires. Subjects who agreed to participate in the present study received a small gift, worth 10RMB ($1.55). The study was approved by the ethics committee of the researcher’s institution, and the procedures and measures were safe for the participants.

### Data analysis

This study used LCA, a person-centered statistical method, to describe the heterogeneity of childhood maltreatment among college students in preschool education. There were no missing data for any of the latent class indicators. Full information maximum likelihood (FIML; Arbuckle, 1996) was used to estimate the small amount (<5%) of missing data for depression and anxiety. All analyses in this study were conducted using Mplus 7 Version 4 (Muthén and Muthén, 2012). All tests were two-tailed for significance, and significance (*p*-value) was set at 0.05.

The subtypes of child maltreatment in this study can be represented by four indicators, including physical abuse, emotional abuse, physical neglect, and emotional neglect with four-point coding (0 = no maltreatment, 1 = low maltreatment, 2 = moderate maltreatment, and 3 = severe maltreatment). These coding methods were based on Bernstein et al. (2003). Specifically, for physical abuse and neglect, if the score was under 7, it was coded as 0, if the score was between 8 and 9, it was coded as 1, if the score was between 10 and 12, it was coded as 2, and if the score was higher than 13, it was coded as 3. For emotional abuse, if the score was under 8, it was coded as 0, if the score was between 9 and 12, it was coded as 1, if the score was between 13 and 15, it was coded as 2, and if the score was higher than 16, it was coded as 3. For emotional abuse, if the score was under 9, it was coded as 0, if the score was between 10 and 14, it was coded as 1, if the score was between 15 and 17, it was coded as 2, and if the score was higher than 18, it was coded as 3 (Bernstein et al., 2003).

Multiple fit indices were used to determine the best-fitting class solution to child maltreatment in this study. The values of the Akaike Information Criterion (AIC; Akaike, 1987), the Bayesian Information Criterion (BIC; Schwarz, 1978), and the adjusted Bayesian Information Criterion (aBIC; Sclove, 1987) were included as comparative fit indices, with lower values of AIC, BIC, and aBIC indicating a relatively better fitting class solution. Moreover, the entropy value was used to measure the reliability of the classification. The entropy value ranged between 0 and 1, and values close to 1 indicated a good classification (Nagin, 2009). Additionally, the Lo–Mendell–Rubin (aLRT) Adjusted Likelihood ratio test was used for class model comparison. A significant aLRT ratio test indicated that an *n* class solution was a significantly better fit than the *n-1* model (Lo et al., 2001).

The role of childhood maltreatment profiles in the relationships between childhood maltreatment and depression and anxiety was investigated based on the optimal LCA class solution. The Bolck-Croon-Hagenaars Method (BCH method; Bolck et al., 2017), was used as the recommended statistical procedure in Mplus for comparing outcome variables across latent classes (Asparouhov and Muthén, 2021). Using the BCH method, measures of depression and anxiety were included as auxiliary variables in the optimal class solution, and means on the above four dimensions were estimated for each class to create the most likely class membership based on item response probabilities.

## Results

### Identifying profiles of childhood maltreatment

Using four categorical indicators (physical and emotional abuse, physical and emotional neglect), one to six profile solutions were considered. The 5-class solution was identified as the best fitting profile solution according to its lowest BIC value, with goodness of fit indices presented in Table 2. Furthermore, compared to the 5-class solution, the 6-class solution had the lowest values of AIC and aBIC; however, it did not perform better than the 5-class solution (*p* > 0.05). Additionally, the entropy for the 5-class solution was 0.98, indicating that the 5 profiles had a clear classification. Thus, the 5-class solution was the best fit for these data.

**Table 2:** Fit information for LCAs modeling maltreatment subtypes (*n* = 1,218).

Classes	LL^a^	AIC	BIC	aBIC	Entropy	aLRT^b^
1	−4273.971	8575.941	8647.411	8602.941		
2	−3254.694	6567.387	6715.431	6623.315	0.998	<0.001
3	−2970.516	6029.032	6253.650	6113.888	1.000	<0.001
4	−2868.859	5855.718	6156.911	5969.503	0.998	<0.001
5	−2789.010	**5726.020**	**6103.787**	**5868.733**	0.984	<0.001
6	−2740.818	5659.636	6113.978	5831.277	0.986	0.3332

^a^ Log likelihood for the class solution. ^b^ *p*-values for the Lo–Mendell–Rubin adjusted likelihood ratio test comparing the n class solution fit with the *n*-1 class solution. The meaning of the bold values is the best solution for class classification selection.

### Characteristics of the 5-class solution

**Figure 1:**
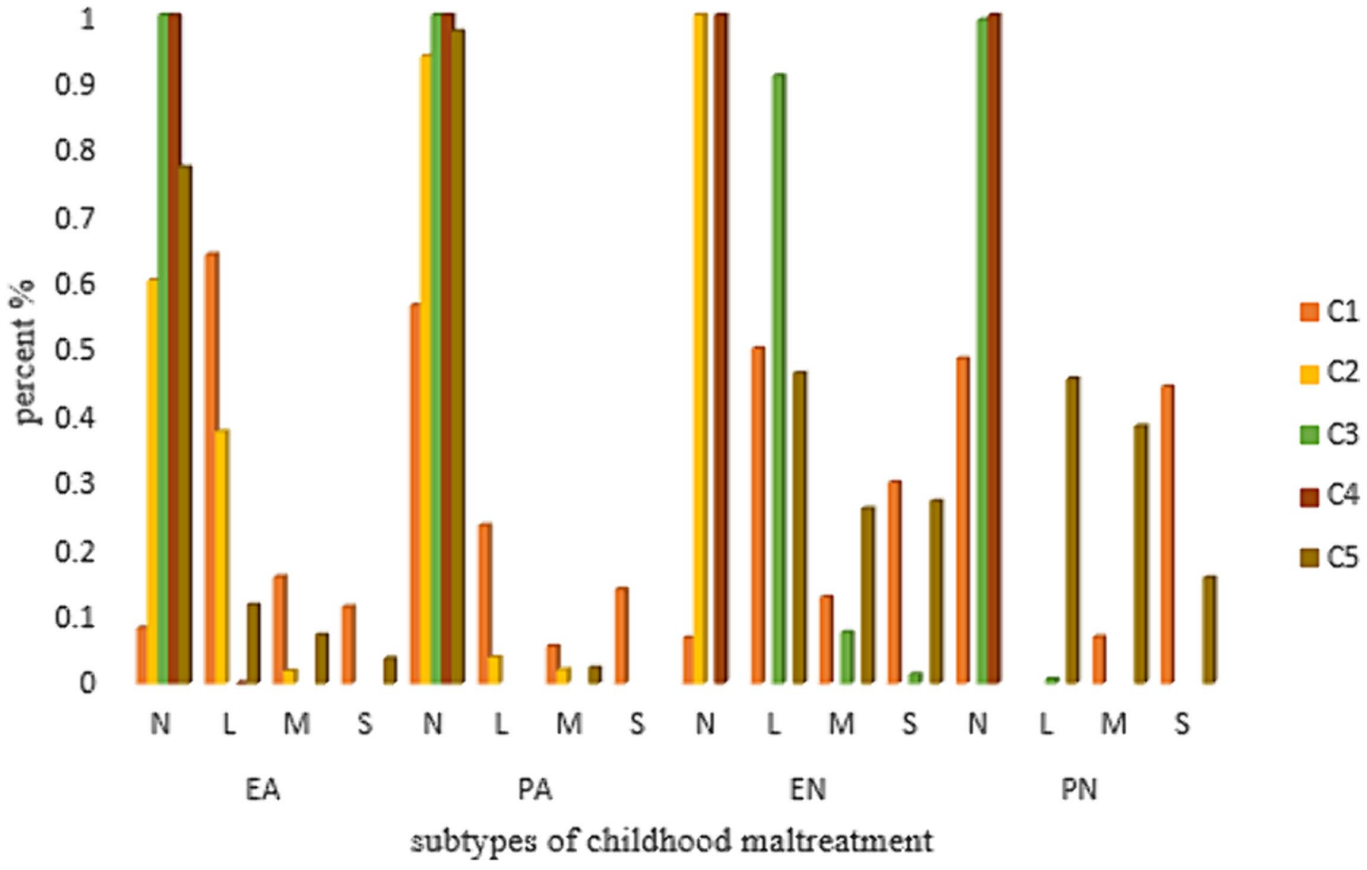
Characteristics of subgroups of childhood maltreatment. EA, emotional abuse; EN, emotional neglect; PN, physical neglect; NM, no maltreatment; PA, physical abuse; L, low; M, moderate; S, severe; C, subtype/profile; C1, EA + EN + PN; C2, PN; C3, EN; C4, NM; C5, EN + PN.

The participants were divided into five profiles, including four maltreated profiles (total prevalence: 44%) and one non-maltreated profile (56%). Within the four maltreated profiles, the emotional and physical neglect profile, characterized by high probabilities of emotional and physical neglect, was the largest maltreatment profile. The second largest maltreatment profile was the emotional neglect profile, which had a high probability of emotional neglect. In addition, the smallest profile was the profile of emotional abuse, and emotional and physical neglect, which characterized by high levels of emotional abuse, emotional neglect, and physical neglect. Detailed information on the characteristics of the profiles can be seen in Table 3 and Figure 1.

**Table 3:** Item response probabilities and class membership proportions for a 5-class solution.

	Class1: EA + EN + PN	Class2: PN	Class3: EN	Class4: NM	Class5: EN+ PN
Overall prevalence (*n* = 1,218)	0.07	0.09	0.12	0.56	0.16
Prevalence among maltreated Children (*n* = 533)	0.17	0.19	0.27	0	0.37

		Overall	Mal.	Class1: EA + EN + PN	Class2: PN	Class3: EN	Class4: NM	Class5: EN+ PN
EA	No	0.860		0.083	0.603	1.000	0.999	0.772
	Low	0.100	0.229	0.642	0.377		0.001	0.118
	Moderate	0.025	0.058	0.160	0.019			0.073
	Severe	0.015	0.034	0.115				0.038
PA	No	0.958		0.566	0.939	1.000	1.000	0.976
	Low	0.021	0.049	0.237	0.040			
	Moderate	0.010	0.023	0.056	0.021			0.024
	Severe	0.011	0.024	0.141				
EN	No	0.652		0.069	1.000		1.000	
	Low	0.219	0.501	0.501		0.909		0.465
	Moderate	0.061	0.139	0.129		0.077		0.262
	Severe	0.068	0.156	0.301		0.014		0.273
PN	No	0.751		0.486	0.420	0.993	1.000	
	Low	0.113	0.259		0.468	0.007		0.456
	Moderate	0.074	0.169	0.070	0.083			0.385
	Severe	0.062	0.141	0.444	0.029			0.159

EA, emotional abuse; EN, emotional neglect; PN, physical neglect; NM, no maltreatment; PA, physical abuse.

### 5-class solution latent profile comparisons for depression and anxiety

The results of the BCH method showed that depression and anxiety differed by class or subgroup of childhood maltreatment. Profile means and differences between profiles on depression and anxiety are presented in Table 4.

**Table 4:** The differences of depression and anxiety based on classifications of childhood maltreatment.

	Class 1: Emotional and mental neglect, physical neglect M (SE)	Class 2: Physical neglect M (SE)	Class 3: Emotional neglect M (SE)	Class 4: No maltreatment M (SE)	Class5: Emotional and physical neglect M (SE)	Pairwise comparisons (*p* < 0.05)
Class prevalence	0.07	0.09	0.12	0.56	0.16	
Depression	2.14 (0.065)	1.88 (0.062)	1.73 (0.040)	1.68 (0.019)	1.90 (0.043)	2, 3, 4, 5 < 1; 3,4 < 2; 3, 4 < 5
Anxiety	1.98 (0.073)	1.83 (0.070)	1.61 (0.041)	1.51 (0.020)	1.71 (0.046)	1 < 3, 4, 5; 2 < 3, 4, 5

Denotes a significant (*p* < 0.05) omnibus Chi-Squared test of group difference between latent class mean values.

Differences between profiles in depression scores were significant (*χ^2^* (4) =70.06, *p* < 0.001). Depression scores in the non-maltreated profile were lower than in the emotional abuse, emotional neglect and physical neglect profiles (*p* < 0.001), the emotional and physical neglect profile (*p* < 0.05), and the physical neglect profile (*p* < 0.01). Moreover, depression scores in the emotional abuse, emotional neglect and physical neglect profile (*p* < 0.001) were higher than in the emotional and physical neglect profile (*p* < 0.01), the physical neglect profile (*p* < 0.01), and the emotional neglect profile (*p* < 0.001).

Differences between profiles in the anxiety score were significant (*χ^2^* (4) =65.00, *p* < 0.001). Anxiety scores in the non-maltreated profile were lower than in the emotional abuse, emotional neglect and physical neglect profiles (*p* < 0.001), the physical neglect profile (*p* < 0.001), the emotional and physical neglect profile (*p* < 0.001) and the emotional neglect profile (*p* < 0.05). Moreover, anxiety scores were higher in the emotional abuse, emotional neglect and physical neglect profiles (*p* < 0.001) than in the emotional and physical neglect profile (*p* < 0.01) and the emotional neglect profile (*p* < 0.001).

## Discussion

This study identified profiles of childhood maltreatment and examined the role of profiles of childhood maltreatment in the associations between childhood maltreatment and depression and anxiety in 1218 Chinese college students in preschool education. This is the first study to explore the role of childhood maltreatment profiles in the associations between childhood maltreatment and later mental health status in this population of college students, which may provide some evidence and suggestions for the training of preschool teachers.

The results show that maltreated preschool education college students were classified into four profiles: emotional abuse, and emotional and physical neglect; physical neglect; emotional neglect; and physical and emotional neglect. These findings are consistent with previous research showing that childhood maltreatment is heterogeneous (Brown et al., 2019; Warmingham et al., 2019). The hierarchy of the parent–child relationship in traditional Chinese culture may influence modern Chinese parents’ beliefs about parenting, which in turn may influence their relationships with their children and contribute to childhood maltreatment. Specifically, the profile of physical and emotional neglect was the largest group in this study, suggesting that a combination of physical and emotional neglect was the most common subtype of childhood maltreatment among college students in preschool education. Traditional Chinese culture emphasizes the parenting beliefs that punishment is one of the most effective ways to discipline children (e.g., Filial sons emerge from under the stick), and these beliefs may raise the possibility of using physical maltreatment during parenting practice. Moreover, Chinese people may be conservative about expressing their feelings, which may contribute to the high prevalence of emotional abuse.

These findings confirm the hypothesis that childhood maltreatment is heterogeneous, suggesting that individual differences should be considered when exploring strategies for preventing childhood maltreatment in a Chinese cultural context. Similarly, these findings describe situations of childhood maltreatment among college students in preschool education, indicating that childhood experiences should be considered an influential factor in the training of Chinese students in preschool education.

Moreover, the results show that childhood maltreatment profiles influenced the associations between childhood maltreatment and depression and anxiety, which was consistent with previous studies (Torchalla et al., 2012). Specifically, individuals belonging to the non-maltreatment profile had lower levels of depression and anxiety than any other maltreatment subgroup, which was in line with other studies (Gardner et al., 2019; Kluczniok et al., 2020). Survivors who experienced childhood maltreatment may have biased perceptions of themselves and others, which may affect later development (e.g., Chen and Qin, 2020a). The results also show that individuals with multiple subtypes of childhood maltreatment had the highest levels of depression and anxiety, suggesting that the accumulation of childhood maltreatment subtypes may increase the level of impact on later development (Russotti et al., 2021).

These findings suggest that mental health was different based on the profiles of childhood maltreatment among Chinese college students in preschool education. They also indicate that we should consider the profiles of childhood maltreatment when exploring the associations between childhood maltreatment and its outcomes. Similarly, these findings also suggest that cumulative childhood maltreatment has a strong impact on later development.

This study can enrich the literature on the profiles of childhood maltreatment, and expand the studies on childhood maltreatment in the context of Chinese culture, in addition to providing some suggestions for the training of preschool teachers. Some limitations should also be acknowledged. First, although this study considered the subtypes, degree, and number of subtypes of childhood maltreatment as indicators, it still may not show the whole picture of child maltreatment. Future studies should add some other aspects of child maltreatment as indicators, such as the timing of child maltreatment, the developmental periods, or the chronicity of child maltreatment. Second, this study included a low number of male pre-service preschool teachers, which ignores gender differences. Future studies should include more male college students in preschool education. Third, this study used depression and anxiety as indicators of mental health, which may not represent all aspects of mental health. Future studies should consider more indicators of mental health when exploring differences in mental health among subgroups of childhood maltreatment, such as emotional stability. Last, this study collected data during the outbreak of COVID-19, which may influence the levels of depression and anxiety (Lee et al., 2024). Future studies may be able to replicate the findings of this study after COVID-19.

Despite the above limitations, this study has several implications. First, this study identified the profiles of childhood maltreatment, suggesting that individual differences may be an important factor for childhood maltreatment studies. Future studies should take individual differences into consideration when exploring childhood maltreatment. Second, this study examined the role of profiles of childhood maltreatment in the associations between childhood maltreatment and mental health status, suggesting that the relationships between adverse childhood experiences and later development may be influenced by the characteristics of childhood maltreatment. Governments should pay much more attention to childhood maltreatment, and to improving the mental health status of individuals. Finally, this study offers important implications for ECE policymakers to pay much more attention to childhood maltreatment when training preschool teachers. Furthermore, this study provides meaningful messages for Chinese preschool managers who seek empirical evidence to improve the quality of preschool teachers. Preschool managers should provide more school-based training aimed at reducing teachers’ depression and anxiety.

## Conclusion

Using latent class analysis based on Chinese preschool education college students, this study identified the profiles of childhood maltreatment and examined the role of profiles of childhood maltreatment in the relationships between childhood maltreatment and depression and anxiety in the Chinese cultural context. The results showed that preschool education college students based on childhood maltreatment were divided into 5 profiles: a non-maltreatment profile, an emotional abuse, emotional, and physical neglect profile, a physical neglect profile, an emotional neglect profile, and a physical and emotional neglect profile. Each profile differed in depression and anxiety, and the emotional abuse, emotional, and physical neglect profile had the greatest impact on depression and anxiety. These findings suggest that childhood maltreatment is heterogeneous and that multiple subtypes of childhood maltreatment strongly influence depression and anxiety. Therefore, it is necessary to consider childhood maltreatment when training preschool teachers.

## Data availability statement

The raw data supporting the conclusions of this article will be made available by the authors, without undue reservation.

## Ethics statement

The studies involving humans were approved by Faculty of Education Beijing Normal University. The studies were conducted in accordance with the local legislation and institutional requirements. The participants provided their written informed consent to participate in this study.

## Author contributions

SJ: Data curation, Data curation, Writing – review & editing. CC: Conceptualization, Project administration, Writing – original draft, Writing – review & editing.
